# Automated analysis of connected speech reveals early biomarkers of Parkinson’s disease in patients with rapid eye movement sleep behaviour disorder

**DOI:** 10.1038/s41598-017-00047-5

**Published:** 2017-02-02

**Authors:** Jan Hlavnička, Roman Čmejla, Tereza Tykalová, Karel Šonka, Evžen Růžička, Jan Rusz

**Affiliations:** 1 Czech Technical University in Prague, Faculty of Electrical Engineering, Department of Circuit Theory, Technická 2, 166 27 Prague 6, Czech Republic; 2 Charles University in Prague, First Faculty of Medicine, Department of Neurology and Centre of Clinical Neuroscience, Kateřinská 30, 120 00 Prague 2, Czech Republic

## Abstract

For generations, the evaluation of speech abnormalities in neurodegenerative disorders such as Parkinson’s disease (PD) has been limited to perceptual tests or user-controlled laboratory analysis based upon rather small samples of human vocalizations. Our study introduces a fully automated method that yields significant features related to respiratory deficits, dysphonia, imprecise articulation and dysrhythmia from acoustic microphone data of natural connected speech for predicting early and distinctive patterns of neurodegeneration. We compared speech recordings of 50 subjects with rapid eye movement sleep behaviour disorder (RBD), 30 newly diagnosed, untreated PD patients and 50 healthy controls, and showed that subliminal parkinsonian speech deficits can be reliably captured even in RBD patients, which are at high risk of developing PD or other synucleinopathies. Thus, automated vocal analysis should soon be able to contribute to screening and diagnostic procedures for prodromal parkinsonian neurodegeneration in natural environments.

## Introduction

As the most complex acquired human motor skill, speech is extremely sensitive to disturbances in the basal ganglia, which are involved in the planning, programming and execution of motor tasks^1–3^. Thus, speech changes are among the most robust motor abnormalities in Parkinson’s disease (PD); a common neurological disorder associated with basal ganglia dysfunction. Up to 90% of PD patients develop perceptually distinctive speech and voice abnormalities, collectively termed hypokinetic dysarthria, characterized by decreased quality of voice, hypokinetic articulation, hypophonia, monopitch, monoloudness and deficits in timing^4,5^. However, previous research has mainly focused on the later stages of PD^5^, while identification of different patterns of vocal disorders in the preclinical course of PD neurodegeneration has been severely restricted^6^. Identifying biomarkers related to neurodegeneration is essential as they could provide invaluable information not only related to prognosis and treatment, but also in the setting of clinical trials and disease modifying therapies before the onset of motor manifestations^7,8^. In this regard, vocal assessment has a potential advantage as an inexpensive, non-invasive and simple-to-administer method, scalable to large populations with the potential to be performed remotely, even by smartphone from the patient’s home.

The development of a fully-automated method to detect early, distinctive patterns of neurodegeneration using only acoustic data from connected speech such as reading the short passage or monologue would have the potential to revolutionize the diagnostic process in neurodegenerative diseases manifesting motor speech disorders. The investigation of prodromal speech changes in subjects with rapid eye movement sleep behaviour disorder (RBD) provides a unique opportunity to evaluate the reliability and utility of such a tool. It is well known that people with RBD are at extremely high risk (>80%) for developing PD and related neurodegenerative disorders^7,8^, and no prodromal disease marker has a predictive value near RBD^9^.

The value of speech assessment in the differential diagnosis of motor speech disorders was suggested half a century ago in the landmark work by Darley *et al.* ^10^, noting that the constellation of specifically affected speech dimensions typically reflects the presumed underlying pathophysiology. Although there has been a substantial increase in computational power in recent years, most of the current methods for the evaluation of dysarthria patterns still rely either on perceptual tests, hand-labelling speech signal or manual control of the analysis procedure^11,12^. Nevertheless, several automated and quantitative approaches based on speech signal processing and machine learning have emerged for the evaluation of speech performance in PD^13,14^. However, these previous methods were designed for highly functional vocal paradigms such as sustained phonation or syllable repetition, and tested on only small samples of PD speakers^13,14^. Currently, no automatic, algorithm-based system is available that would allow robust and sensitive evaluation of different natural, connected speech patterns across a wide range of disease severity, from non-perceptible preclinical speech changes to the dysarthria in PD patients.

We developed a fully automated speech monitoring system that uses a segmentation method for the precise estimation of voiced and unvoiced segments of speech, respirations and pauses. We further proposed a set of acoustic speech features based on the segmentation algorithm applicable to connected speech, allowing the description of complex vocal disturbances due to neurodegeneration including respiratory deficits, dysphonia, imprecise articulation and dysrhythmia. We show that subliminal speech abnormalities can be reliably captured even in RBD patients and thus consider automated analysis suitable for research on vocal development in PD with potential clinical implications.

## Results

### Data collection

Typical vocal deficits associated with PD were examined using sample of 30 patients with newly diagnosed, untreated PD as compared to 50 healthy subjects without any history of neurological or communication disorders. Subsequently, we collected sample of 50 subjects with idiopathic RBD in order to reveal speech alterations that are typical for prodromal PD (see Table 1 and Methods). All participants were asked to perform two speaking tasks that represent natural speech and reflect motor speech disorders comprehensively^5^. First, speakers read a standardized, phonetically-balanced text of 80 words twice (Supplementary Fig. S1). Second, participants were instructed to provide monologue about their interests, job, family or current activities for approximately 90 seconds. Both PD and RBD subjects were scored according to the Unified Parkinson’s Disease Rating Scale motor subscore (UPDRS III^15^, ranging from 0 to 108, with 0 for no motor manifestations and 108 representing severe motor distortion). The severity of dysarthria in PD and RBD individuals was perceptually described by speech item 18 of the UPDRS III.

**Table 1:** Clinical characteristics of newly diagnosed, untreated PD patients and RBD subjects.

	PD (n = 30)	RBD (n = 50)
Mean Age (years)	64.9 (SD 10.9, range 34–79)	64.9 (SD 9.1, range 40–83)
Men	70% (n = 21)	82% (n = 41)
Women	30% (n = 9)	18% (n = 9)
Positive history of Parkinson’s disease in family	7% (n = 2)	2% (n = 1)
Mean age of disease onset (years)	63.4 (SD 11.9, range 30–78)	59.2 (SD 9.8, range 33–81)
Age of disease onset <40 years	10% (n = 3)	4% (n = 2)
Mean symptoms duration (years)	1.6 (SD 1.3, range 0.5–6)	5.8 (SD 4.4, range 1–17)
Mean Hoehn & Yahr score	2.1 (SD 0.3, range 1.5–2.5)	n/a
Mean UPDRS III total	20.2 (SD 12.4, range 6–54)	5.2 (SD 4.1, range 0–21)
Mean UPDRS III 18 speech item	0.4 (SD 0.5, range 0–1)	0.06 (SD 0.24, range 0–1)
Antidepressant therapy	10% (n = 3)	14% (n = 7)
Antiparkinsonian therapy	0 (n = 0)	0 (n = 0)
Levodopa equivalent (mg/day)	0	0
Clonazepam therapy	10% (n = 3)	12% (n = 6)
Clonazepam (mg/day)	0.07 (SD 0.22, range 0–1)	0.08 (SD 0.30, range 0–2)

Captions: PD = Parkinson’s disease, RBD = rapid eye movement sleep behaviour disorder, SD = standard deviation, n/a = not applicable.

### Automatic segmentation of connected speech

**Figure 1:**
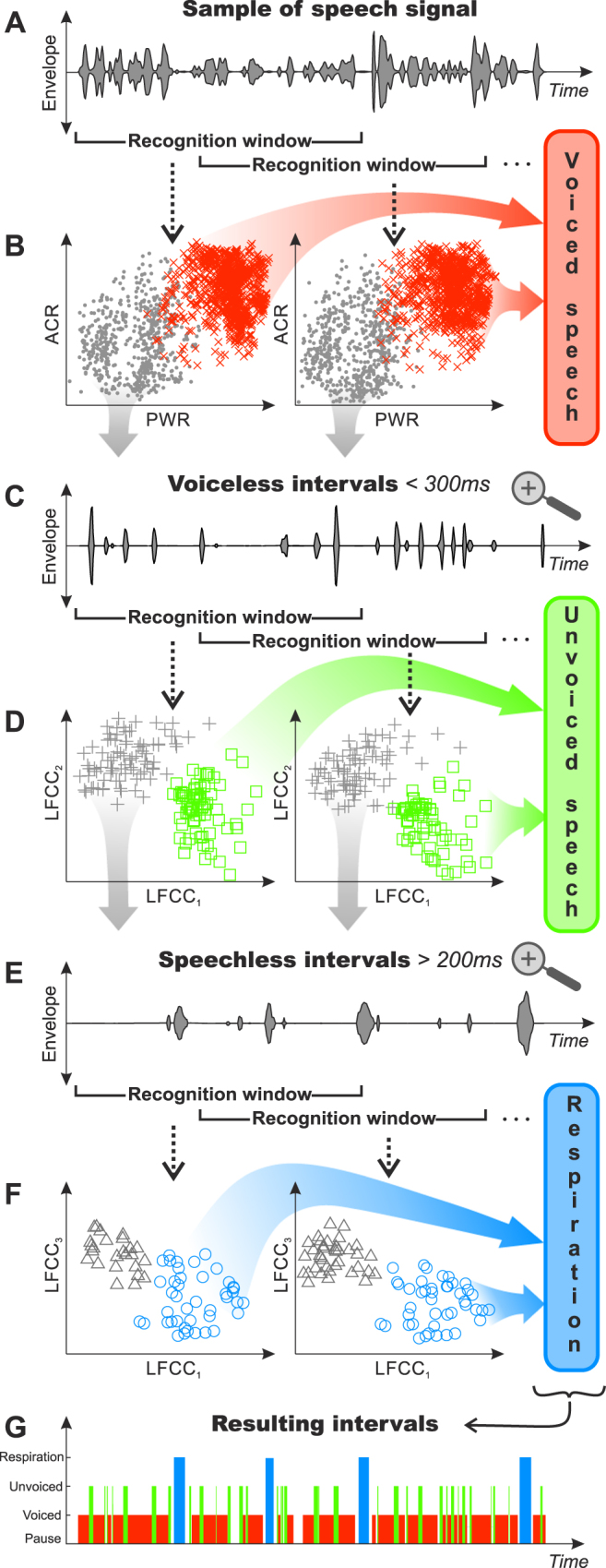
Process diagram illustrating the principle of the automated segmentation algorithm on a speech signal sample. (**A**) Original speech sample depicted using oscillographic sound pressure plot. (**B**) Red ‘x’ marks illustrate recognized voiced speech depicted in dimensions of PWR and ACR in two consecutive recognition windows. (**C**) Speech sample representing voiceless intervals shorter than 300 ms after removal of voiced intervals from original speech sample. (**D**) Green ‘o’ marks illustrate recognized unvoiced speech depicted in dimensions of the first linear-frequency cepstral coefficient (LFCC_1_) and the second linear-frequency cepstral coefficient (LFCC_2_) in two consecutive recognition windows. (**E**) Speech sample representing respiration intervals longer than 200 ms after removal of voiced and unvoiced intervals from original speech sample. (**F**) Blue ‘o’ marks illustrate recognized respirations depicted in dimensions of the first linear-frequency cepstral coefficient (LFCC_1_) and the third linear-frequency cepstral coefficient (LFCC_3_) in two consecutive recognition windows. (**G**) Resulting intervals of the segmentation plotted in time. PWR = power, ACR = variance of autocorrelation function.

The main challenge of the proposed algorithm consisted in the class-by-class recognition of four basic physiological sources of connected speech including voiced speech, unvoiced speech, pause and respiration (see Methods, Fig. 1, Supplementary Fig. S2, and Supplementary Movie S1). To make the segmentation adaptive, the speech signal was processed inside a recognition window, where the signal was further parameterized by a sliding window into zero-crossing rate (ZCR), variance of autocorrelation function (ACR), power (PWR) and linear-frequency cepstral coefficients (LFCC). Voiced speech was determined using a cluster analysis in the space of ZCR, ACR and PWR. Subsequently, unvoiced speech was recognized using a cluster analysis in the space of the first five LFCC using voiceless intervals shorter than 300 ms in order to avoid misclassification with respirations. Consecutively, respirations were determined by a cluster analysis in the space of the first five LFCC using speechless intervals longer than 200 ms associated with the approximate minimal duration required for inspiration. Finally, the resulting intervals were described by time labels for voiced speech, unvoiced speech, pause and respiration, where pauses include all respirations.

### Tracking method performance

To compare and evaluate the reliability of the proposed segmentation method, manual reference labels were introduced for pause and respiration intervals. A total of 200 randomly chosen recordings including both speaking tasks across each group of participants was labelled blindly without awareness of segmentation output using speech analysis software^16^. Both pauses and respirations were labelled with respect to speech context using predefined criteria based on visual inspection of the spectrogram (see Methods).

The performance of the segmentation algorithm was evaluated for pause and respiration detection independently using hand labels. In addition, the performance of pause detection was compared with a previously designed pause detector for dysarthric speech^17^ as well as a voice activity detector of ITU-T G.729B^18^ commonly used in telecommunications for the reduction of transmission rate during silent periods of speech (see Methods).

The proposed segmentation method showed superior performance of 86.2 ± 7.5% efficiency across all pause lengths, in comparison to 55.4 ± 9.6% obtained using the previously designed pause detector for dysarthric speech^17^, and 33.9 ± 6.2% by the voice activity detector^18^. As short pauses are less likely to be determined correctly by hand-labelling and long pauses play an important role in speech production, final performances of all detectors are described by the cumulative distribution of the mean detection efficiency depending on the duration of pause (Supplementary Fig. S3A). The pauses longer than approximately 100 ms are difficult to detect due to occurrence of respiratory signals that share similar characteristics with certain unvoiced fricatives (e.g. velar fricatives). Pauses shorter than approximately 100 ms are challenging to detect because non-speech turbulent airflow occurring during a pause can excite a spectrum similar to that of the preceding phoneme from articulators. In addition, insufficiently articulated unvoiced consonants can be hidden in natural noise background.

The proposed segmentation of respiration achieved 81.6 ± 15.2% efficiency through all respiration durations. Efficiency of respiration is expressed as the cumulative distribution of mean detection efficiency as a function of the duration of respiration (Supplementary Fig. S3B).

### Acoustic speech features

**Figure 2:**
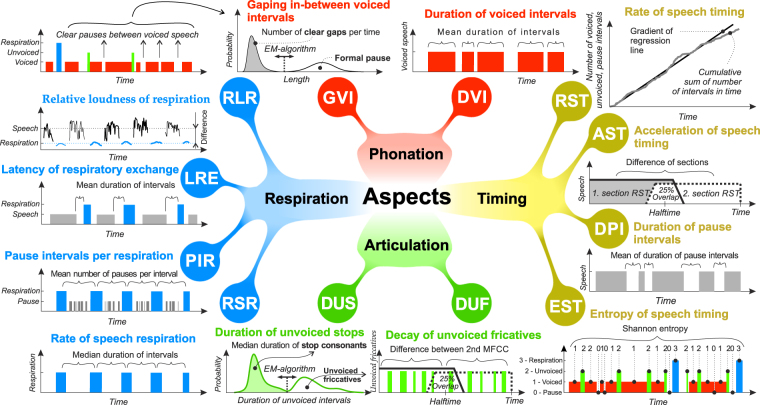
Mind map illustrating basic principles of individual acoustic speech features. MFCC = Mel-frequency cepstral coefficients.

Based on the outcome of adaptive segmentation, we designed a set of 12 acoustic features that were utilized with respect to speech disturbances associated with PD^5^, allowing the assessment of all basic subsystems of connected speech including timing, articulation, phonation and respiration (see Methods, Fig. 2, and Supplementary Table S1).

Timing features involve information about the rhythmic organization of speech. We evaluated the speech rate with respect to quality of speech timing as the rate of speech timing (RST) including voiced, unvoiced and pause intervals. Acceleration of speech associated with parkinsonism was computed by acceleration of speech timing (AST) including voiced, unvoiced and pause intervals. Duration of pause intervals (DPI) describes the quality of speech timing, as pauses can be heavily influenced by the ability to properly initiate speech. Heterogeneity of speech in terms of the occurrence of voiced, unvoiced, pause and respiratory intervals was measured by entropy of speech timing (EST).

The assessment of articulation was quantified on intervals of unvoiced speech that represent pure involvement of the supra-laryngeal muscles. Performance of the most challenging articulatory movements represented by stop consonants was directly measured by the duration of unvoiced stops (DUS). In addition, the temporal quality of articulation was determined from unvoiced fricatives using the decay of unvoiced fricatives (DUF).

Phonatory characteristics were evaluated using intervals of voiced speech. The fundamental phonatory measurement was then the mean duration of voiced intervals (DVI). Deficits of phonatory onset and offset control were measured by gaping in-between voiced speech (GVI).

The respiratory apparatus was evaluated using data from detected respiratory intervals principally representing inspirations. The rate of speech respiration (RSR) robustly estimates respiratory rate during speech. Breath groups were described using pause intervals per respiration (PIR). The relative loudness of respiration (RLR) evaluates audibility of respiration relative to loudness of speech, eliminating dependence on microphone gain. The latency of respiratory exchange (LRE) measures the pause between expiration represented by the time speech ends and respective inspiration.

**Figure 3:**
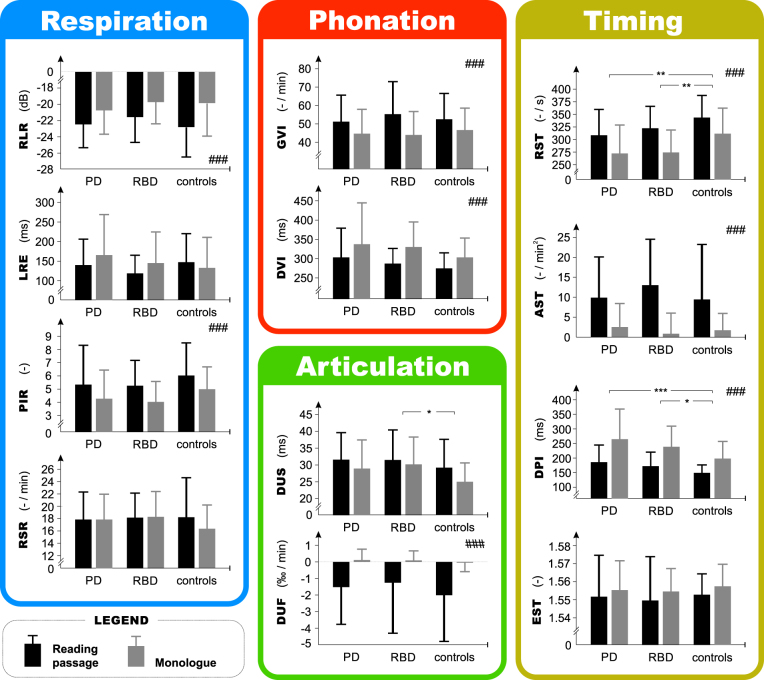
Results of acoustic speech analyses. Bars represent mean values and error bars represent SD values. Repeated measures analysis of variance (RM-ANOVA) was used to test for group differences: GROUP (PD vs. RBD vs. controls): corrected *p < 0.05, **p < 0.01, ***p < 0.001 after Bonferroni adjustment; TASK (reading passage vs. monologue): corrected ^#^ *p* < 0.05, ^##^ *p* < 0.01, ^###^ *p* < 0.001 after Bonferroni adjustment. None of the features showed significant GROUP × TASK interaction. RST = rate of speech timing, AST = acceleration of speech timing, DPI = duration of pause intervals, EST = entropy of speech timing, DUS = duration of unvoiced stops, DUF = decay of unvoiced fricatives, DVI = duration of voiced intervals, GVI = gaping in-between voiced intervals, RSR = rate of speech respiration, PIR = pause intervals per respiration, RLR = relative loudness of respiration, LRE = latency of respiratory exchange, PD = Parkinson’s disease, RBD = rapid eye movement sleep behaviour disorder.

Forty-three percent of PD patients and only 6% of RBD subjects perceptually demonstrated mildly affected speech (score of 1) according to the UPDRS III speech item; 57% of PD patients and 94% of RBD subjects showed normal speech (score of 0). However, significant group differences were found for RST and DPI speech timing features, primarily associated with both differences between PD and control groups as well as between RBD and control groups (Fig. 3). Articulatory feature of DUS also significantly discriminated investigated groups, mainly due to the significant differences between RBD and control groups but also due to observed trend between PD and control groups (*p* = 0.03, uncorrected). All features except EST, DUS, RSR and LRE showed significant differences between reading passage and monologue (Fig. 3 ). No significant correlations were observed between any of the speech features and UPDRS III.

### Index test

**Figure 4:**
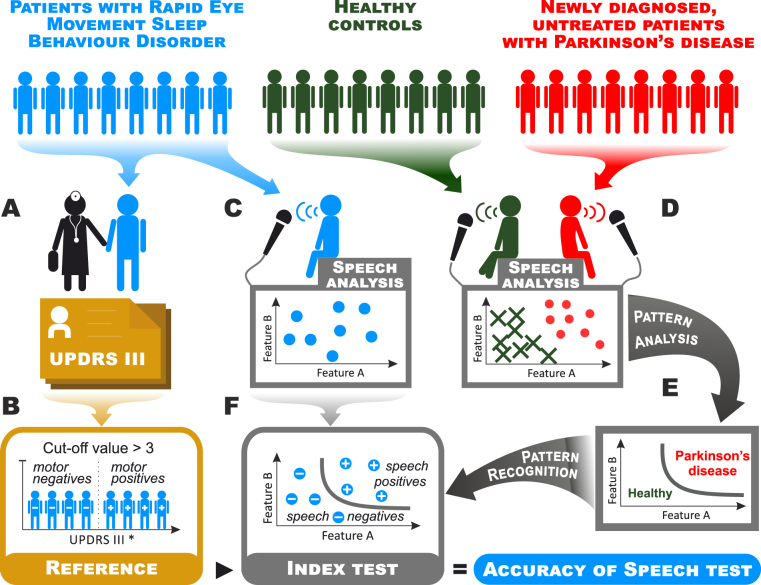
Flowchart describing the procedure of speech test experiment. (**A**) The motor score of UPDRS was examined in each RBD subject by a well-trained neurologist. (**B**) RBD subjects were separated into a subgroup of *motor negatives* (asymptomatic, UPDRS III* ≤ 3) and a subgroup of *motor positives* (symptomatic, UPDRS III* > 3). (**C,D**) Speech performances of all newly diagnosed, untreated PD patients, RBD subjects, and controls were analyzed using the set of designed speech features. (**E**) The most distinctive parkinsonian speech patterns were determined as the best resulting combination of speech features for differentiating between newly diagnosed, untreated PD patients and controls. (**F**) All RBD subjects were separated into a subgroup of *speech positives* (subjects with unchanged speech performance) and a subgroup of *speech negatives* (subjects with speech performance closer to PD speakers) based on the most distinctive parkinsonian speech pattern obtained through comparison between newly diagnosed, untreated PD and controls. PD = Parkinson’s disease, RBD = rapid eye movement sleep behaviour disorder, UPDRS = Unified Parkinson’s Disease Rating Scale; UPDRS III* = motor part of the UPDRS III score after removal of action tremor.

A blinded experiment was designed using UPDRS III with removal of action tremor to improve its predictive value^7^ (hereafter UPDRS III*) (Fig. 4A), which is common in non-parkinsonian conditions^7^. UPDRS III* at a cut-off > 3 was previously revealed to be a very good indicator of initial parkinsonism^7^, and thus RBD subjects with UPDRS III* ≤ 3 (hereafter asymptomatic RBD subgroup) were labelled as *motor negatives* and RBD subjects with UPDRS III* > 3 (hereafter symptomatic RBD subgroup) were labelled as *motor positives* (Fig. 4B). The speech performances of all RBD speakers were analysed automatically without any supervision (Fig. 4C). As PD patients show dysarthria profiles with unequal severity and different types of predominant speech manifestations^4,19^, the speech pattern associated with PD was determined by sequential selection through all extracted speech features (Fig. 4D). This experiment was executed by quadratic discriminant function analysis, where each combination of features was evaluated using a leave-one-subject-out cross-validation scheme as follows: One individual speaker was excluded from the dataset iteratively, and the multivariate normal distribution characterized by the mean and covariance matrix was estimated using an Expectation Maximization algorithm for PD and controls independently. The excluded speaker was assigned to the distribution of PD or controls using Bayes discriminant rule. The best combination of acoustic features representing the most salient speech pattern of PD was determined as the one with highest accuracy (Fig. 4E). Finally, all RBD subjects were classified using the resulting speech pattern learned from the dataset of PD and control subjects (Fig. 4F). Speech performances of all RBD subjects assigned to the distribution of PD were labelled as *speech positives*, otherwise *speech negatives*. Finally, we obtained the true positive score as the number of *motor positives* equal to *speech positives*, the true negative score as the number of *motor negatives* equal to *speech negatives*, the false positive score as the number of *motor negatives* equal to *speech positives*, and the false negative score as the number of *motor positives* equal to *speech negatives*. Discriminative efficiency was then described by accuracy, sensitivity, and specificity.

**Figure 5:**
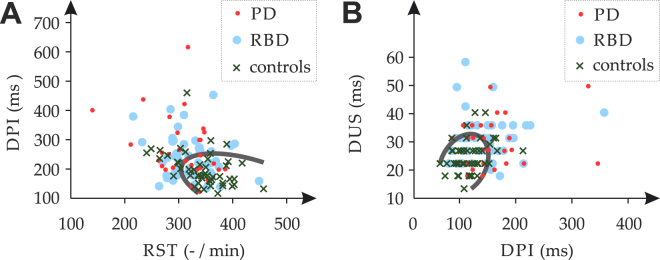
Selected pairs of representative acoustic features depicting the most distinctive speech pattern allowing differentiation between PD and controls. Individual speakers plotted in speech dimensions represented by (**A**) DPI in the monologue and RST in reading task, and (**B**) DPI in the reading task and DUS in monologue. The solid gray line represents the border of discrimination between *speech negatives* (subjects with unchanged speech performance) and *speech positives* (subjects with speech performance closer to PD speakers). The ‘.’ marks represent PD, ‘o’ for RBD and ‘x’ for controls. PD = Parkinson’s disease, RBD = rapid eye movement sleep behaviour disorder, DPI = duration of pause intervals, RST = rate of speech intervals, DUS = duration of unvoiced stops.

As a result, a subgroup of 27 asymptomatic RBD subjects was classified using *motor negatives* labels, whereas a subgroup of 23 symptomatic RBD subjects was classified using *motor positives* labels. The most distinctive disturbed speech patterns between the PD and control groups were found for a combination of RST in reading passage, DVI in monologue, DPI in reading passage, DPI in monologue, DUS in reading passage, DUS in monologue and PIR in monologue with 71.3% accuracy (56.7% sensitivity and 80.0% specificity). Based upon the predictive model obtained through comparison between PD and controls, the results of the speech test indicate that *motor positives* and *motor negatives* from the RBD group were determined with 70.0% accuracy (73.9% sensitivity and 66.7% specificity) (Fig. 5).

## Discussion

The results of our work represent the first step toward the development of a fully automated tool for the large-scale evaluation of prodromal vocal impairment due to neurodegeneration. Our findings indicate that the time for basic research through automated quantitative vocal analysis of natural speech is already upon us. The newly designed algorithm has proven sufficiently transparent to provide suggestions on typical patterns of parkinsonian prodromal vocalization deficits in RBD. Thus, the present study demonstrates the potential benefit of adding objective acoustic evaluation to the standard test battery used to identify those at high risk of developing neurodegeneration^20^.

Interestingly, although the vast majority of RBD subjects were without perceptible speech impairment as indicated by the UPDRS III speech item, we objectively captured similar speech timing abnormalities in both de novo PD and RBD subjects. Indeed, converging evidence from neuroimaging, limb control and neuropsychological studies has suggested the presence of timing deficits in PD due to the inability to maintain a programmed response or to rapidly switch between responses^21,22^. Dysrhythmia patterns in our PD and RBD cohort included prolongation of pauses and decreased rate of speech intervals. While the prolongation of pauses in PD has been well documented^23^, the decreased rate of speech intervals revealed in the present work provides new insight into the production of speech in PD. In particular, decreased rate of speech intervals indicates less diversity between follow-up speech segments, likely as a result of decreased range of motion of the speech apparatus. On closer examination there were also trends toward changes in measurement of voiced intervals duration, representing voicing leakage through pauses, with mean values for the RBD group intermediate between PD patients and controls. Thus, the decreased rate of speech intervals and prolonged pauses appear to also be, at least partially, influenced by a decreased ability to stop voicing properly, which may reflect weak adduction of the vocal folds due to bradykinesia and rigidity of laryngeal muscles. In addition to the decreased ability to stop voicing properly, RBD subjects tend to extensively pronounce unvoiced stop consonants to acoustically resemble fricatives due to insufficiently closed articulators, which can be considered a precursor of the phenomenon called spirantization^24^. These observations are in agreement with previous research suggesting that PD with RBD may represent a distinct phenotype manifesting more as an akinetic-rigid syndrome, in comparison to PD without RBD^25,26^.

In accordance with the majority of previous studies^27^, we did not observe direct correlations between speech and motor assessment in PD and RBD. Nevertheless, we were able to blindly predict symptomatic motor RBD group membership with 70% accuracy based on speech assessment. In general, we may thus assume that speech impairment partially parallels increasing limb motor disability due to the underlying neurodegenerative process. Considering that motor dysfunction strongly predicts disease onset, regardless of primary diagnosis of parkinsonism or dementia^7,20^, the screening of motor speech changes may improve stratification for future neuroprotective therapy against PD and other synucleinopathies.

Automated segmentation methods for connected speech in dysarthria such as the speech-pause detector are very rare. One might assume that the precise identification of pauses is an easy task as it is used on a daily basis for voice activity detection in telecommunication transmission. However, the efficiency of detection was only 33.9% using a traditional voice activity detector^18^, as it requires detection in the condition of environmental noise, and thus most non-speech sounds including respiration are accepted as voice activity. In particular, efficiency of detection was not substantially improved even using a previously designed pause detector for dysarthric speech^17^, which reached an accuracy of only 55.4%. However, this method is based on thresholding the power envelope and thus will always struggle with any presence of non-speech sounds and the turbulent airflow of insufficiently closed articulators in general^17^. The pause detection of our algorithm reached a very high accuracy of 86.2% and substantially outperformed conventional methods. Additionally, although no respiration detector suitable for dysarthria evaluation is currently available, the proposed segmentation method also achieved high efficiency of 81.6% for the detection of respirations.

Our proposed algorithm is applicable for the assessment of complex parkinsonian vocal deficits and will likely be able to track speech progression as we found significant differences between controls and mild to moderate PD with more extended treatment periods across all speech subsystems including timing, articulation, phonation and respiration (Supplementary Fig. S4). In addition, developed algorithm also proved to be suitable for evaluation of both types of common connected speech including reading passage and monologue. While during the reading task the speaker is simply pronouncing ready-made text and thus can provide attention to articulatory planning, spontaneous speech requires more linguistic planning and the ability to formulate thoughts, allowing the speaker to modify the rhythm of his speech to breathe more freely^28^. Indeed, our findings indicate that most speech dimensions were influenced by the type of speaking task and both tasks can provide useful information. Although we did not find any interaction between task and group effects across speech dimensions, we still may assume that the temporal measurements such as speech acceleration and articulation decay are related to the content of speech and thus are better suited for a reading task as was previously shown^29^. It is also prudent to consider the effect of language and gender on speech characteristics. As the natural rhythm of the speech differs across languages^30^, the specific speech dimensions designed in the present study cannot be simply transcribed to other languages, but need to be slightly rescaled with respect to common speech performances of healthy speakers of the given language. Interestingly, previous research has also suggested that gender may have a confounding impact on the progression of specific speech impairment in PD due to sexual dimorphism of laryngeal size^31^. In agreement with previous studies reporting a clearly increased incidence of PD in men^32^ and a strong male predominance for RBD of up to 90%^33^, our PD and RBD groups also consisted mainly of male participants and thus we cannot exclude the possibility that certain speech patterns may be influenced by gender-specific aspects. We should also point out that we did not stratify our PD patients according to the presence of RBD symptoms^23^, as we wanted to test our algorithm using a representative sample of PD-related dysarthric speech that could be influenced by various factors such as disease phenotype. Finally, as the primary aim of the current study was to develop an automated monitoring system allowing the assessment of connected speech, we validated our findings based upon the reference to neurological evaluation using UPDRS III and did not evaluate any neuroimaging biomarkers that could support our findings. Recently, it has been shown that methods such as resting state functional magnetic resonance imaging or single-photon emission computed tomography scanning may provide sensitive indicators of early basal ganglia dysfunction^34,35^. Future studies are needed to evaluate our findings in other languages and in relation to other suitable biomarkers sensitive to prodromal neurodegeneration due to PD and other synucleinopathies.

In conclusion, our results indicate that the automated analysis of thoughtfully-selected acoustic features with well-defined pathophysiology from recordings of connected speech can be a reliable tool for monitoring vocalization deficits associated with neurodegeneration based on pathological alpha-synuclein storage, from non-perceptible preclinical to more advanced dysarthria stages. We believe that the procedure can be further elaborated and translated into other languages as well as to the entire spectrum of neurodegenerative disorders manifesting motor speech disorders. The current pilot findings provide novel opportunities for future research on motor speech disorders ranging from traditional laboratory-based analyses, monitoring the effect of therapy and disease progression, to the possibility of high-throughput screening for prodromal neurodegeneration, followed by more detailed analysis if the screen is abnormal.

## Methods

### Data collection

From 2014 to 2016, a total of 130 Czech native speakers were recruited for the study. For a given large effect size (Cohen’s f of 0.4), we determined a minimum sample size of 84 with at least 30 per group by power analysis^36^, with the error probability (α) set at 0.05 and a false negative rate (β) set at 0.1 (i.e. power of 0.9), based on a 3-group RM-ANOVA with one covariate (GROUP). Each participant provided written, informed consent and the study was approved by the Ethics Committee of the General University Hospital in Prague, Czech Republic (approval number 67/14 Grant VES AZV 1. LFUK). The study was carried out in accordance with the approved guidelines. Thirty patients (21 men, 9 women) with de novo, untreated PD, mean age 64.9 (standard deviation [SD] 10.9), were diagnosed upon the Parkinson’s Disease Society Bank Criteria^37^ (Table 1). In addition, 50 subjects (41 men, 9 women), mean age 64.9 (SD 9.1), were diagnosed with idiopathic RBD according to the International Classification of Sleep disorders diagnostic criteria, third edition^38^ (Table 1). As a control group, 50 healthy subjects (41 men, 9 women), mean age 63.4 (SD 10.8) years, without a history of neurological or communication disorders, were included in the study.

The study was carried out at a single center. All PD patients were consecutively recruited at their first visit to the clinic and were examined in the drug-naive state, before symptomatic treatment was started. RBD subjects were screened through a web-based online survey^39^. No RBD patient complained of motor or cognitive difficulties or had a history of treatment with antiparkinsonian medication or any other therapy influencing sleep, cognition or motor features. Both diagnosis and evaluation of clinical scales were performed by a well-trained professional neurologist with experience in movement disorders. As the diagnosis of individual PD or RBD subjects was made at evaluation, the specific date recorded for each participant was different, but overall time schedule was the same. Each participant was first scored clinically by the neurologist and subsequently examined during a single session with a speech specialist. Speech data were recorded in a quiet room with a low ambient noise level using a head-mounted condenser microphone (Bayerdynamic Opus 55, Heilbronn, Germany) situated approximately 5 cm from the mouth. Recordings were sampled at 48 kHz with 16-bit resolution. None of the participants underwent speech therapy before the investigation.

### Automatic segmentation of connected speech

All samples were preprocessed, and parameterization was established. Subsequently, each speech signal was modelled using a Gaussian mixture model (GMM), representing the most common method used in speech signal processing applications. Estimating all groups at once is not effective because all mixtures are separated imperfectly in single parametric space and false-positive errors as well as false-negative errors might occur. However, precise and robust classification is ensured if individual speech classes are estimated sequentially with respect to the corresponding traits in most differenced parameters. Sequential separation was executed via unique recognition steps where each recognition step separated previous distributions into two fractions (Supplementary Fig. S2).

#### Preprocessing

The signal was decimated to a sampling rate of 8 kHz, which is sufficient for speech recognition. Signal filtering using a 4^th^-order high-pass Chebyshev filter was performed to remove frequencies lower than 130 Hz, which include main hum, popping, and other subsonic sounds. Such a high cut-off frequency did not affect recognition but highlights the voiced speech.

#### Parameterization

The signal was parameterized inside a sliding window of 15 ms, in steps of 5 ms. The PWR, ACR and ZCR were calculated using the following equations:

1 $${\rm{PWR}}=\frac{1}{N}\sum _{n=1}^{N}{x}^{2}[n]\cdot h[n],$$

2 $${R}_{x}[k]=\frac{1}{N\cdot {\sigma }_{x}^{2}}\sum _{n=1}^{N}(x[n]-{\mu }_{x})\cdot (x[n+k]-{\mu }_{x}),$$

3 $${\rm{ACR}}=\frac{1}{N-1}\sum _{k=1}^{N}({R}_{x}[k]-{\bar{R}}_{x}),$$

4 $${\rm{ZCR}}=\frac{1}{N-1}\sum _{n=1}^{N-1}|\mathrm{sign}({R}_{x}[n+1])-\mathrm{sign}({R}_{x}[n])|,$$

5 $$\mathrm{sign}(x[n])=\left\{\begin{matrix}1, & x[n]\ge 0\\ -1, & x[n]< 0\end{matrix}\right.,$$

where *x* is a signal in window of length *N, h* is hamming window, *R*_*x*_ represents the autocorrelation function, *σ*_*x*_ is the standard deviation of the signal, and *µ*_*x*_ is the mean of the signal. Because all parameters can by described by a lognormal distribution, we expressed them on a logarithmical scale. ZCR was computed using the normalized autocorrelation function. This approach emphasizes harmonic frequency and suppresses the noise component of voiced consonants. Thus, all voiced phonemes (vowels and voiced consonants) can be described simply by a unimodal normal distribution of voiced speech, which boosts the sensitivity of detection of voiced speech. ACR was calculated as the variance of the normalized autocorrelation function of the unweighted signal. Signal was also parameterized in the spectrum using the first five of 24 LFCC, representing the low-frequency envelope of the power spectral density.

#### Sequential separation

The principle of sequential separation consisted in step-by-step recognition of the most differentiated components of speech (Supplementary Fig. S2). Speech was separated in the following order: voiced speech (Supplementary Fig. S2A ), unvoiced speech (Supplementary Fig. S2B ), and respiration (Supplementary Fig. S2C ). The recognition step was executed inside a sliding recognition window. As a result, adaptability to the speech apparatus and environmental noise was achieved over time during speech recording. The GMM of parameters were presupposed in each position of the recognition window. GMM actually involve an unpredictable differentiation of mixtures. Therefore, the number of mixtures was evaluated using the Calinski-Harabasz index over the range <*2*; *3*>. The optimal number of mixtures corresponded to a higher index of evaluation. GMM parameters were estimated using the EM-algorithm. Observations were classified via Bayes discriminant rule. Decisions were additionally smoothed by presupposing an Indo-European language family, in which unvoiced speech is accompanied by voiced speech. The values of thresholds of decision smoothing were not settled to exact value but only to approximate the natural timing of the speech apparatus.

#### Voiced speech

Voiced speech was separated from the whole signal using the parametric space of PWR, ACR, and ZCR within a recognition window of 20 seconds in 6 seconds steps. Voiced speech was identified as the component with the highest mean PWR. Decisions were smoothed using a median filter of the 5^th^ order and the following decision rules: voiced segments shorter than 30 ms were classified as voiceless but voiceless segments shorter than 20 ms were classified back as voiced, as such a short-term control over vocal fold function is hardly possible.

#### Unvoiced speech

Unvoiced speech represented by unvoiced consonants was separated from unvoiced segments shorter than 300 ms, which included most consonants and excluded most respiration signals. The first five LFCC were used for recognizing unvoiced consonants. The recognition window was 60 seconds long and featured 20 seconds steps. The prolongation of the recognition window compensated for the low occurrence of consonants. Unvoiced speech was identified as the component with the highest mean of the first LFCC, which is related to loudness. Unvoiced speech shorter than 5 ms and unvoiced speech in distance to voiced speech longer than 30 ms were rejected.

#### Pause

Pauses were defined as unvoiced and non-consonant signals, including the time required for respiration. The minimum duration of pauses was considered to be 30 ms.

#### Respiration

Respirations were separated from residual segments (excluding voiced speech and consonant segments already classified in the first two steps) longer than 200 ms using the first five LFCC. Respirations were identified as the component with the highest mean of the first LFCC. Respirations shorter than 40 ms were rejected. Inspirations were bounded by silence as the lungs reversed the direction of airflow. Therefore, respirations in distance to voiced speech shorter than 30 ms were classified as unvoiced speech. Gaps between respirations shorter than 400 ms were classified as respirations.

#### Labels

Each segment was described using labels pertaining to start time, end time, and class. All signal processing and data analysis steps were done in © Matlab (MathWorks).

### Reference hand labels

The following criteria for pause annotations were derived from Fisher and Goberman^40^:

1. A signal interval can be annotated as a pause only if it contains no harmonic spectrum or noise exceeding the noise floor and exhibiting no formant structure similar to that of speech. Pauses can contain respirations isolated from speech or speech artefacts unrelated to the content of speech.
2. Pause preceding speech ends at time of the origin of formant structure accompanied by a harmonic spectrum or noise signal. If speech is initiated by an explosive consonant, then a pause ends at the time of an initial burst of energy.
3. Pause following speech begins at the time of the breaking of the formant structure associated with the previous phoneme.

The following criteria for respiration annotations were established:

1. Respirations were identified as noise substantially exceeding the noise floor with characteristic resonance within the 500–2000 Hz frequency band in pauses longer than 100 ms.
2. Borders of respiration were identified as the time of highest spectral change between pause and respiratory signals.
3. Non-continuous signals of respiration were labelled as homogenous respiration.

All labels were perceptually verified. Labels were described using time of interval start and end, as well as flag of segment origin including pause or respiration.

### Algorithm performance evaluation

The gold standard for testing was based on reference hand labels, whereas a tolerance field was assigned to each label. Detected labels lying within the tolerance field were interpreted as true positives (TP). Each hand label was paired with only one detection. Unpaired reference hand labels were interpreted as false negatives (FN). Unpaired detections were interpreted as false positives (FP). The efficiency of detection F was evaluated by the F-score:

6 $${\rm{precision}}=\frac{{\rm{TP}}}{{\rm{TP}}+{\rm{FP}}},$$

7 $${\rm{recall}}=\frac{{\rm{TP}}}{{\rm{TP}}+{\rm{FN}}},$$

8 $${\rm{F}}={\rm{2}}\cdot \frac{{\rm{precision}}\cdot {\rm{recall}}}{{\rm{precision}}+{\rm{recall}}}.$$

The tolerance field of pause was obtained around each label, with bounds corresponding to a quarter of the duration of the corresponding pause. The most interesting information pertaining to the segmentation efficiency was the dependence on pause length. Therefore, efficiency was iteratively computed across pauses longer than the progressive threshold from 50 ms to 300 ms in 50 ms steps. Respirations were evaluated within the tolerance field of duration of the corresponding respiration around each label.

The pause detector for dysarthric speech^17^ was set to obtain the best performance result using a 200 bin histogram, a sampling rate of 8 kHz, and same preprocessing procedure used in the currently proposed segmentation method. The voice activity detection^18^ was performed using a 8 kHz sampling rate and standard settings.

### Acoustic speech features processing

To prevent distortion caused by pauses lasting more than several seconds, all pauses longer than 2 seconds were saturated to a maximal duration of 2 seconds.

RST provides a more robust estimate of speech rate impairment than a simple pause rate measurement as it considers not only pause but both voiced and unvoiced intervals. Voiced intervals provide additional information about impairment of phonatory control, whereas unvoiced intervals about imprecise articulation. The rate, including voiced, unvoiced, and pause intervals, approximates the speech rate complexly, as the speech rate impairment is related to deficits in all dimensions of speech. Each voiced, unvoiced, and pause interval was described by the time of occurrence, determined as the mean time between speech interval start and interval end. The total number of intervals was counted for each moment over the course of measurement. RST was computed as the gradient of the regression line of the time course.

AST determines the extent of timing acceleration. Speech run was split into two halftimes with 25% overlap, which provided a smooth transition between parts. AST was computed as the difference between RSTs of both parts divided by the total duration of the sentence.

DPI evaluates a speaker’s ability to initiate speech. Complex speech impairment can cause difficulties in initiating speech, which cause prolongation of pauses. DPI was computed as the median duration of all pause intervals.

EST describes the orderliness or predictability of speech including voiced, unvoiced, pause, and respiratory intervals. Impaired speech associated with hypokinetic dysarthria tends to be more ordered and predictable, as voiced intervals dominate speech at the expense of other types of intervals. Accordingly, decreased entropy is tantamount to impaired speech. Each interval of speech was taken as one observation. Number of all intervals of speech were computed, including number of voiced speech intervals *nv*, number of unvoiced speech intervals *nu*, number of pause intervals *np*, number of respiration intervals *nr*, and total number of intervals *nt*. EST was determined as follows:

9 $${\rm{EST}}=-\,\frac{nv}{nt}\cdot {\mathrm{log}}_{2}\,(\frac{nv}{nt})-\frac{nu}{nt}\cdot {\mathrm{log}}_{2}\,(\frac{nu}{nt})-\frac{np}{nt}\cdot {\mathrm{log}}_{2}\,(\frac{np}{nt})-\frac{nr}{nt}\cdot {\mathrm{log}}_{2}\,(\frac{nr}{nt}).$$

DUS assesses imprecise articulation manifested by increased noise accompanying stop consonants or even by continuant articulation perceived as fricative. Thus, the duration of detected stop consonants tends to increase. The length of unvoiced consonants forms a bimodal GMM of unvoiced fricative consonants and unvoiced stop consonants. This distinctly varied mixture was recognized using the EM-algorithm. DUS was computed as the median duration of recognized intervals of stop consonants.

DUF measures the temporal quality of articulation. Unvoiced fricatives are characterized by energy concentrated at high frequencies (>2.5 kHz). Information about temporal quality of articulation was measured as temporal damping of the high-frequency bulk. Speech run was split into two halftimes with 25% overlap, which provided a smooth transition between parts. Fricative consonants were recognized in each halftime from bimodal GMM of unvoiced fricative consonants and unvoiced stop consonants using the EM-algorithm. Unvoiced fricatives of each halftime were parameterized using 24 Mel-frequency cepstral coefficients (MFCC). The second MFCC is related to the ratio between energies of the high and low Mel-frequency bands. DUF was computed as the mean of differences between the second MFCC of both halftimes weighted on total duration of speech. DUF was expressed in parts per thousand with the respect to the presumed small range of values.

DVI evaluates the phonatory apparatus with respect to neglecting unvoiced consonants and pauses, voicing during voiceless consonants and simple fusing of multiple roots in word or fusing of independent words. DVI was computed as the mean duration of voiced intervals.

GVI represents the measurement of speaker ability to split voiced segments by pauses. Pauses between voiced intervals refer to the separation of different roots of words, independent words, and sentences in general. Impairment of the phonatory apparatus causes fusing of different roots of words or independent words. Pauses associated with unvoiced speech or respiration were rejected. The length of those clear pauses between voiced segments were described by a bimodal normal distribution. Formal pauses and clear gaps were recognized using the EM-algorithm. GVI was computed as the number of clear gaps per total time of speech.

RSR estimates the breathing rate from detected intervals of respiration. The computation of RSR was designed to be robust against misdetections of respiratory intervals. Each respiration was described by the mean time between respiration start and respiration end. The speed of respiration was determined as the inverted median value of this interval expressed in minutes.

PIR aims to examine respiratory and prosodic function in a context characterized by pause production during a breath group. Commonly, a decrease in breath group together with a decrease in pause rate anticipate decreased PIR due to dysarthria. The number of pauses framed between two respirations was taken into account. PIR was computed as the median number of pauses per respiration.

RLR represents a simple loudness measurement for estimating inspiratory effort. Every obstruction in the respiratory path produces noise. The loudness of inspiratory noise is proportional to inspiratory flow and the resistance of obstruction in the respiratory pathway. Limited chest wall kinematics associated with dysarthria reduce inspiratory flow and noise. The loudness of respiratory noise was referenced to the loudness of speech to make the value of RLR independent of microphone gain. The signal was squared and filtered by a moving average of 120 order and rescaled into loudness using a logarithmical scale. RLR was determined as the difference between the median loudness of respirations and the median loudness of speech.

LRE indicates the ability to convert expiration during speech into inspiration. The impaired ability of respiratory exchange manifests in the prolongation of the interval between expiration and inspiration, which must be minimized in speech breathing. Each respiration was evaluated by the latency time, which was computed as the difference between the time of inspiration and the time of expiration. The time of expiration end was determined from the end of the nearest preceding speech interval. The time of inspiration start was determined from the start of the interval of respective respiration. LRE was computed as the mean of latency times of all detected respirations.

### Statistical analysis

For every subject, the final values of 12 acoustic features related to reading passage were computed by averaging the data obtained in two vocal task runs. As all acoustic features were found to be normally distributed by the one-sample Kolmogorov-Smirnov test, statistical analyses were performed using repeated measures analysis of variance with GROUP (PD vs. RBD vs. controls) treated as a between-group factor and TASK (reading passage vs. monologue) treated as a within-group factor. Post-hoc GROUP significance was assessed with the Fisher least-squares difference. Pearson correlations were applied to test for significant relationships. Bonferroni correction for multiple comparisons was applied according to the 12 tests performed with corrected *p* threshold = 0.0042 (i.e., 0.05/12) for *p* < 0.05.

## Electronic supplementary material

Supplementary Material

Supplementary movie S1

Supplementary Dataset 1

## References

[1] Kent RD, Kent JF, Weismer G, Duffy JR (2000). What dysarthrias can tell us about the neural control of speech. J. Phonetics.

[2] Graber S, Hertrich I, Daum I, Spieker S, Ackermann H (2002). Speech perception deficits in Parkinson’s disease: Underestimation of time intervals compromises identification of durational phonetic contrasts. Brain Lang..

[3] Ho AK, Bradshaw J, Iansek R, Alfredson R (1999). Speech volume regulation in Parkinson’s disease: Effects of implicit cues and explicit instructions. Neuropsychologia.

[4] Ho AK, Iansek R, Marigliani C, Bradshaw JL, Gates S (1998). Speech impairment in a large sample of patients with Parkinson’s disease. Behav. Neurol..

[5] Duffy, J. R. Motor Speech Disorders: Substrates, Differential Diagnosis and Management, 3rd ed., Mosby, St. Louis (2013).

[6] Rusz J (2016). Quantitative assessment of motor speech abnormalities in idiopathic REM sleep behaviour disorder. Sleep Med..

[7] Postuma RB, Lang AE, Gagnon JF, Pelletier A, Montplaisir JY (2012). How does Parkinsonism start? Prodromal Parkinsonism motor changes in idiopathic REM sleep behaviour disorder. Brain.

[8] Schenck CH (2013). Rapid eye movement sleep behaviour disorder: devising controlled active treatment studies for symptomatic and neuroprotective therapy-a consensus statement from the International Rapid Eye Movement Sleep Behaviour Disorder Study Group. Sleep Med..

[9] Postuma RB (2012). Identifying prodromal Parkinson’s disease: Pre‐Motor disorders in Parkinson’s disease. Mov. Disord.

[10] Darley FL, Aronson AE, Brown JR (1969). Differential diagnostic patterns of dysarthria. J. Speech Lang. Hear. Res..

[11] Sussman JE, Tjaden K (2012). Perceptual measures of speech from individuals with Parkinson’s disease and multiple sclerosis: Intelligibility and beyond. J. Speech Lang. Hear. Res..

[12] Skodda, S., Grönheit, W., Mancinelli, N. & Schlegel, U. Progression of voice and speech impairment in the course of Parkinson’s disease: a longitudinal study. *Parkinsons Dis**.* **389195** (2013). 10.1155/2013/389195 PMC3872441 24386590

[13] Little MA, McSharry PE, Hunter EJ, Spielman J, Ramig LO (2009). Suitability of dysphonia measurement for telemonitoring of Parkinson’s disease. IEEE T. Biomed. Eng.

[14] Novotný M, Rusz J, Čmejla R, Růžička E (2014). Automatic evaluation of articulatory disorders in Parkinson’s disease. IEEE/ACM T. Audio Speech Lang. Proces.

[15] Stebbins GT, Goetz CG (1998). Factor structure of the Unified Parkinson’s Disease Rating Scale: motor examination section. Mov. Disord.

[16] Boersma P (2001). Praat, a system for doing phonetics by computer. Glot International.

[17] Rosen K (2010). Automatic method of pause measurement for normal and dysarthric speech. Clin. Linguist. Phonet..

[18] International Telecommunication Union. Standardization Sector of ITU. ITU-T G.729, WTSC-96, Geneva, 1–39 (1996).

[19] Rusz J, Čmejla R, Růžičková H, Růžička E (2011). Quantitative acoustic measurements for characterization of speech and voice disorders in early untreated Parkinson’s disease. J. Acoust. Soc. Am..

[20] Postuma RB, Gagnon JF, Bertrand JA, Marchand DG, Montplaisir JY (2015). Parkinson risk in idiopathic REM sleep behavior disorder Preparing for neuroprotective trials. Neurology.

[21] Benecke J, Rothwell C, Dick PJR, Day PL, Marsden CD (1987). Disturbance of sequential movements in patients with Parkinson’s disease. Brain.

[22] Spencer KA, Rogers MA (2005). Speech motor programming in hypokinetic and ataxic dysarthria. Brain Lang..

[23] Hammen VL, Yorkston KM (1996). Speech and pause characteristics following speech rate reduction in hypokinetic dysarthria. J. Commun. Disord..

[24] Weismer, G. & McNeil, M. Articulatory characteristics of Parkinsonian dysarthria: Segmental and phrase-level timing, spirantization, and glottal-supraglottal coordination. The dysarthrias: Physiology, acoustics, perception, management, College-Hill Press, San Diego 101–130 (1984).

[25] Romenets SR (2012). Rapid eye movement sleep behavior disorder and subtypes of Parkinson’s disease. Mov. Disord.

[26] Gong Y (2014). Clinical manifestations of Parkinson disease and the onset of rapid eye movement sleep behavior disorder. Sleep Med..

[27] Sapir S (2014). Multiple factors are involved in the dysarthria associated with Parkinson’s disease: a review with implications for clinical practice and research. J. Speech Lang. Hear. Res..

[28] Levelt WJM (1989). Speaking: From Intention to Articulation. A Bradford Book.

[29] Skodda S, Schlegel U (2008). Speech rate and rhythm in Parkinson’s disease. Mov. Disord.

[30] Ramus F, Nespor M, Mehler J (1999). Correlates of linguistic rhythm in the speech signal. Cognition.

[31] Hertrich I, Ackermann H (1995). Gender-specific vocal dysfunctions in Parkinson’s disease: electroglottographic and acoustic analyses. Ann. Oto. Rhinol. Laryn.

[32] de Lau LM, Breteler MM (2006). Epidemiology of Parkinson’s disease. Lancet Neurol..

[33] Trotti LM (2010). REM sleep behaviour disorder in older individuals. Drugs Aging.

[34] Rolinski M (2016). Basal ganglia dysfunction in idiopathic REM sleep behaviour disorder parallels that in early Parkinson’s disease. Brain.

[35] Berg D (2015). MDS research criteria for prodromal Parkinson’s disease. Mov Disord.

[36] Faul F, Erdfelder E, Lang A-G, Buchner A (2007). G*Power 3: A flexible statistical power analysis program for the social, behavioral, and biomedical sciences. Behav. Res. Methods.

[37] Hughes AJ, Daniel SE, Kilford L, Lees AJ (1992). Accuracy of clinical diagnosis of idiopathic Parkinson’s disease: a clinico-pathological study of 100 cases. J. Neurol. Neurosurg. Psychiatry.

[38] American Academy of Sleep Medicine. International Classification of Sleep Disorders, Third Edition: Diagnostic and Coding Manual, Westchester, Illinois: American Academy of Sleep Medicine (2014).

[39] Buskova, J., Ibarburu, V., Sonka, K. & Ruzicka, E. Screening for REM sleep behavior disorder in the general population. *Sleep Med.* **24**, 147 (2016). 10.1016/j.sleep.2016.07.003 27697450

[40] Fischer E, Goberman AM (2010). Voice onset time in Parkinson disease. J. Commun. Disord..

