# Supplementary material for: Automated analysis of connected speech reveals early biomarkers of Parkinson’s disease in patients with rapid eye movement sleep behaviour disorder
Source: Sci Rep. 2017 Feb 2;7:12. doi: 10.1038/s41598-017-00047-5 (PMC5428345; doi:10.1038/s41598-017-00047-5)
Supplement: Supplementary file 1 — Supplementary Material [file 41598_2017_47_MOESM1_ESM.pdf]

Supplementary Information

Automated analysis of connected speech reveals early biomarkers of Parkinson's disease in patients with rapid eye movement sleep behaviour disorder

Jan Hlavnička[1], Roman Čmejla[1], Tereza Tykalová[1], Karel Šonka[2], Evžen Růžička[2], Jan Rusz[1,2*]

[1]Czech Technical University in Prague, Faculty of Electrical Engineering, Department of Circuit Theory, Technická 2, 166 27, Prague 6, Czech Republic
[2]Charles University in Prague, First Faculty of Medicine, Department of Neurology and Centre of Clinical Neuroscience, Kateřinská 30, 120 00, Prague 2, Czech Republic

Supplementary Figures and Table

"Když člověk poprvé vsadí do země sazeničku, chodí se na ni dívat třikrát denně: tak co, povyrostla už nebo ne? I tají dech, naklání se nad ní, přitlačí trochu půdu u jejích kořínků, načechrává jí lístky a vůbec ji obtěžuje různým konáním, které považuje za užitečnou péči. A když se sazenička přesto ujme a roste jako z vody, tu člověk žasne nad tímto divem přírody, má pocit čehosi jako zázraku a považuje to za jeden ze svých největších osobních úspěchů."

Supplementary Figure S1: Standardized, phonetically-balanced Czech text of 80 words.

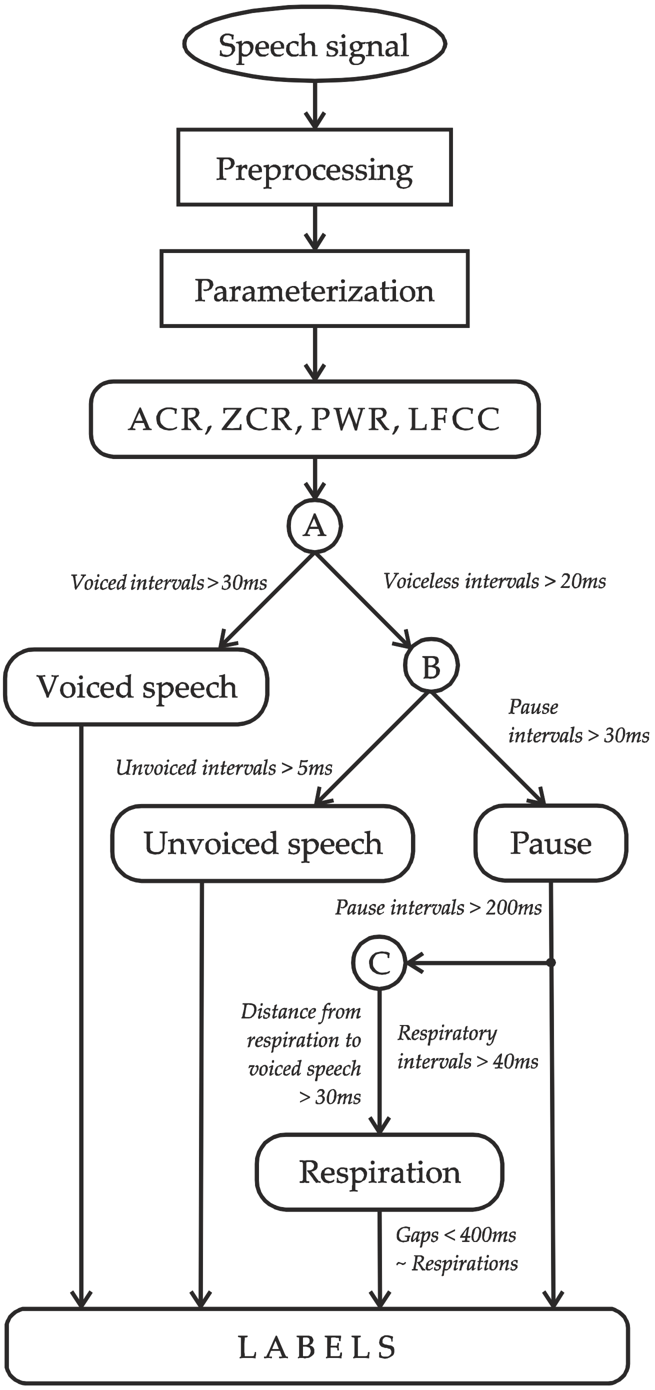

Supplementary Figure S2: Flow chart of automated algorithm describes full process of segmentation of speech signal in basic physiological sources of signal, including voiced speech, unvoiced speech, pause, and respiration. The signal was decimated and high-pass-filtered in a preprocessing step. Subsequently, parameterization was performed and the parametric space of PWR, ACR, ZCR, and LFCC was sequentially separated in a given order: (A) Voiced speech was separated. (B) Unvoiced speech and pause were separated out of the remaining set. (C) Respirations were detected in pauses. PWR = power, ACR = variance of autocorrelation function, ZCR = zero-crossings-rate, and LFCC = linear frequency cepstral coefficients

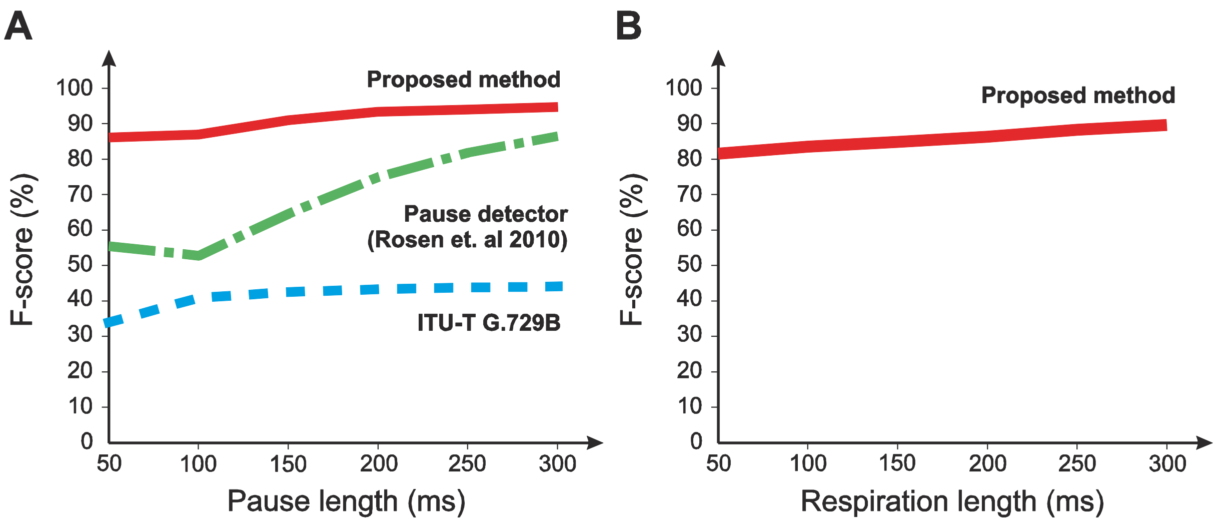

Supplementary Figure S3: Evaluation of automated segmentation of pause and respiration. (A) Performance comparison of proposed automated segmentation method for pauses (red line) with pause detector for dysarthric speech (green line) and VAD ITU-T G.729B algorithm (blue line). (B) Performance of proposed automated segmentation method for respiration. VAD = voice activity detection

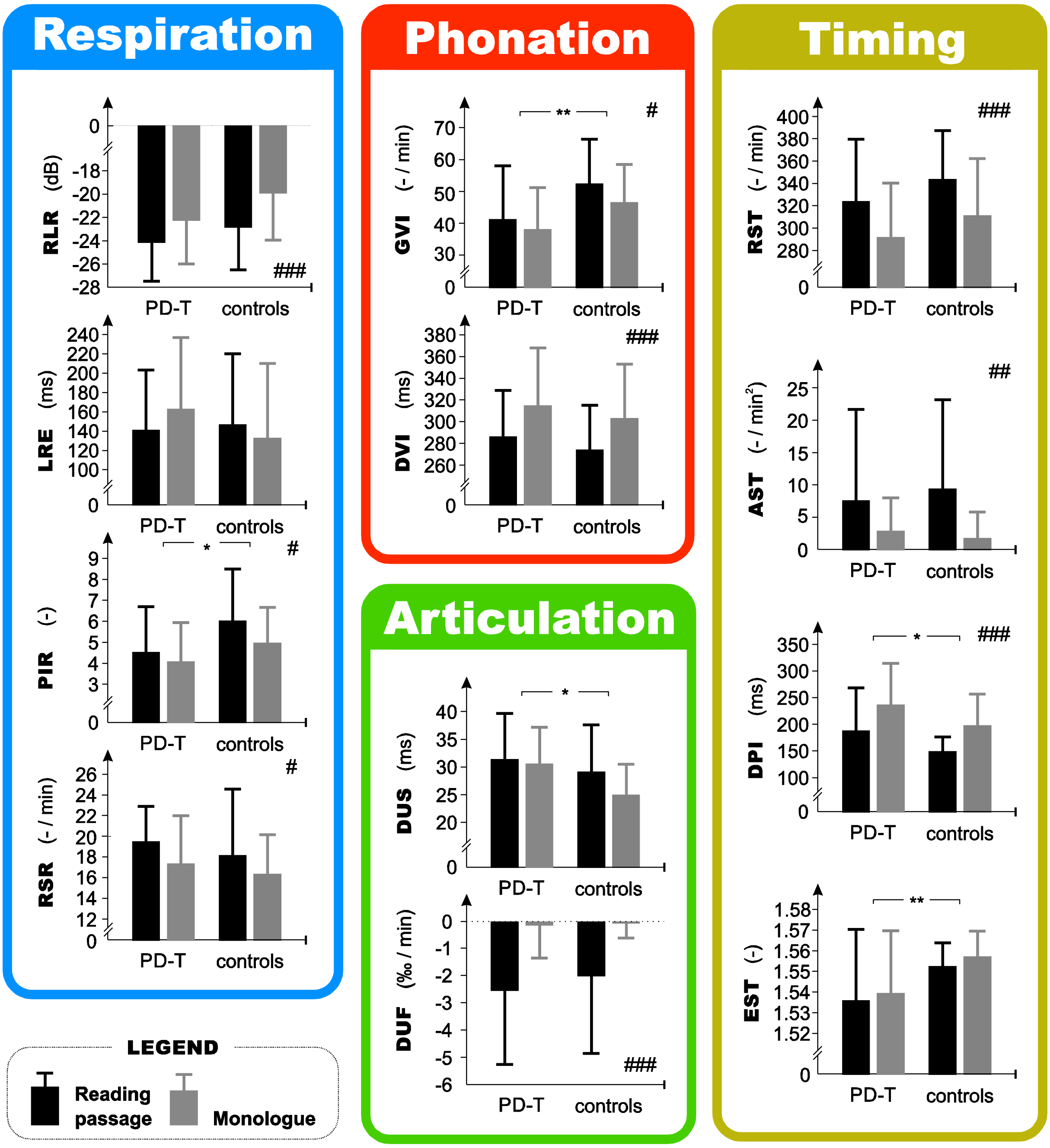

Supplementary Figure S4: Results of acoustic speech analyses between healthy controls (n=50; description of dataset can be found in main text) and patients with mild to moderate PD treated with levodopa (hereafter PD-T, n=40). The mean age of PD-T group (23 men, 17 women) was 64.0 (SD 9.7) years, the mean duration of PD symptoms prior to examination was 7.0 (SD 3.1) years, the mean Hoehn & Yahr score was 2.2 (SD 0.4), the mean UPDRS III score was 18.5 (SD 8.8) and the mean UPDRS III speech item was 0.9 (SD 0.6). All PD-T subjects were on stable dopaminergic medication for at least 4 weeks prior to the examination with a mean levodopa equivalent dose of 762 (SD 365) mg/day. Bars represent mean values and error bars represent SD values. Repeated measures analysis of variance (RM-ANOVA) was used to test for group differences: GROUP (PD vs. controls): corrected * $p<0.05$, ** $p<0.01$, *** $p<0.001$ after Bonferroni adjustment; TASK (reading passage vs. monologue): corrected $^{\#}$ $p<0.05$, $^{\#\#}$ $p<0.01$, $^{\#\#\#}$ $p<0.001$ after Bonferroni adjustment. None of the features showed significant GROUP x TASK interaction.

Figure S4 captions: RST = rate of speech timing, AST = acceleration of speech timing, DPI = duration of pause intervals, EST = entropy of speech timing, DUS = duration of unvoiced stops, DUF = decay of unvoiced fricatives, DVI = duration of voiced intervals, GVI = gaping in-between voiced intervals, RSR = rate of speech respiration, PIR = pause intervals per respiration, RLR = relative loudness of respiration, LRE = latency of respiratory exchange, PD = Parkinson’s disease.

Supplementary Table S1: Overview of applied speech measurements.

Feature name	Abbreviation	Definition	Pathophysiological interpretation with respect to dysarthria
**Timing**			
Rate of speech timing	RST	Rate of voiced, unvoiced and pause intervals measured as the slope of the regression line of total interval count per time. Each interval was described as mean time between onset and offset of interval.	Hypokinetic movements of speech apparatus lead to reduced stream of voiced, unvoiced and pause intervals.
Acceleration of speech timing	AST	Mean difference between RST of a segment parted in two halves with 25% overlap.	Acceleration of speech rate is achieved at the expense of reduced RST.
Duration of pause intervals	DPI	Median length of pause intervals.	Hypokinesia of speech apparatus makes initiating of speech difficult, leading to prolonged pause intervals.
Entropy of speech timing	EST	Shannon information entropy was computed from the frequency of voiced, unvoiced, pause and respiration intervals.	Increased orderliness and predictability of pathological speech results in decreased entropy and lower variation of timing.
**Articulation**			
Duration of unvoiced stops	DUS	Median length of unvoiced stop consonants identified from the bimodal distribution of length of unvoiced stop consonants and unvoiced fricatives using an Expectation Maximization algorithm.	Period of stop consonants is prolonged by friction-like noise of insufficiently closed articulators.
Decay of unvoiced fricatives	DUF	Mean difference between the second Mel-frequency cepstral coefficients, associated with the ratio between energies of low and high Mel-frequency bands, of unvoiced fricatives weighted on squared duration of speech which was divided in two halves with 25% overlap.	Temporal decrease of range of articulatory movement is manifested by loss of high-frequency energy in unvoiced fricatives.
**Phonation**			
Duration of voiced intervals	DVI	Mean length of voiced intervals.	Incomplete or unperformed closure of vocal folds leads to longer voiced intervals and voicing leakage through inter and intra-word pauses.
Gaping in-between voiced intervals	GVI	Rate of clear pauses between voiced intervals. Clear pause is a gap between two voiced intervals containing no consonant or respiration. Formal pauses were excluded from the bimodal distribution of length of clear pauses using an Expectation Maximization algorithm.	Deteriorated ability to properly stop vocal fold vibration.
**Respiration**			
Rate of speech respiration	RSR	Number of respirations per unit time.	Rigidity of respiratory muscles, respiratory dyskinesia or posture issues are related to increased respiratory rate.
Pause intervals per respiration	PIR	Median number of pauses between respirations.	Impaired ability to stop respiratory airflow manifests as decreased pause production.
Relative loudness of respiration	RLR	Median of loudness measured relatively between respirations and speech as difference in logarithmic scale.	Hypokinesia of respiratory muscles and decreased range of rib cage motion make respiration quieter.
Latency of respiration exchange	LRE	Mean duration between end of speech and start of respiration.	Rigidity and bradykinesia of respiratory muscles causes higher latency of exchange between expiration and inspiration.

Supplementary Movie S1: Animation demonstrating outcome of automated separation of connected speech into four basic physiological sources including voiced speech, unvoiced speech, pause, and respiration for a representative healthy control and Parkinson’s disease speaker.
